# Increased risk for alcohol- and other substance-exposed pregnancies among women who smoke tobacco: A secondary analysis of a primary care-based intervention

**DOI:** 10.18332/tid/191107

**Published:** 2024-07-26

**Authors:** Thomas F. Northrup, Angela L. Stotts, Stephen M. Fischer, Kirk L. von Sternberg, Mary M. Velasquez

**Affiliations:** 1 Department of Family and Community Medicine, University of Texas Health Science Center at Houston, McGovern Medical School, Houston, United States; 2 Department of Psychiatry and Behavioral Sciences, UTHealth Houston, McGovern Medical School, Houston, United States; 3 Health Behavior Research and Training Institute, University of Texas at Austin, Austin, United States

**Keywords:** substance-exposed pregnancy, preconception, alcohol-exposed pregnancy, tobacco-exposed pregnancy

## Abstract

**INTRODUCTION:**

Among women at risk for alcohol-exposed pregnancies (AEP), smoking tobacco may be associated with increased severity of alcohol use, and risk for tobacco-exposed and other substance-exposed pregnancies (TEPs/SEPs). Our secondary data analysis of the ‘CHOICES Plus’ intervention trial explored AEP and SEP risk by smoking status.

**METHODS:**

Eligible women (N=261) were recruited from 12 primary care clinics in a public healthcare system, not pregnant, aged 18–44 years, drinking >3 drinks/day or >7 drinks/week, sexually active, and not using effective contraception. We compared women who did and did not smoke tobacco on alcohol and drug severity, and psychological distress (e.g. anxiety) at baseline.

**RESULTS:**

Participants were primarily Hispanic (47.1%) or non-Hispanic Black (41.8%) and reported incomes <$20000/year (69.3%). Tobacco smoking prevalence was 45.2%. Compared to non-smokers, those who smoked drank more days/week (mean=3.3, SD=2.0 vs mean=2.7, SD=1.8, p<0.01), had higher alcohol use disorders identification test (AUDIT) scores (mean=12.1, SD=7.6 vs mean=9.8, SD=7.1, p<0.05), were more likely to report current drug use (66.1% vs 48.3%, p<0.01), and had a greater number of (lifetime) drugs used (mean=3.0, SD=2.0 vs mean=2.0, SD=1.5 days, p<0.0001). Also, those who smoked reported greater levels of anxiety (mean=5.9, SD=5.6 vs mean=4.5, SD=4.9, p<0.05), lower confidence to not drink (mean=2.8, SD=0.8 vs mean=3.1, SD=1.0, p<0.01), lower confidence to reduce risky drinking (mean=6.3, SD=3.1 vs mean=7.3, SD=2.8, p<0.0001), greater drinking temptations (mean=3.0, SD=0.9 vs mean=2.6, SD=0.9, p<0.01), and, yet greater readiness to reduce alcohol use (mean=6.2, SD=3.0 vs mean=5.2, SD=3.0, p<0.05).

**CONCLUSIONS:**

Women who drink and smoke may have the highest AEP, TEP, and other SEP risk. Primary care providers should screen for alcohol and tobacco co-use and provide brief intervention and/or treatment referral.

**CLINICAL TRIAL REGISTRATION:**

The study was registered on the official website of ClinicalTrials.gov

**IDENTIFIER:**

ID NCT01032772

## INTRODUCTION

Approximately one-third (35.7%) of pregnancies in the US are unintended^1^, and the proportion may be even greater among people using alcohol^2^ or tobacco^3^. Many pregnant women may not know they are pregnant and/or delay prenatal care, potentially exposing their unborn fetuses to lengthier periods and potentially greater quantities of tobacco, alcohol, and other substance exposures^4^. The preconception period is especially important for helping sexually active people who may become pregnant to learn of their risks for alcohol and other substance exposure during pregnancy to minimize risks.

Among sexually active people who are not using effective contraception during vaginal intercourse, and are at risk for alcohol-exposed pregnancies (AEP), smoking tobacco may be associated with higher levels of alcohol use severity and increased risk for other substance-exposed pregnancies (SEPs). Tobacco smoking is associated with co-use of alcohol among pregnant people and people who may become pregnant^5^; however, this association and the potential for increased pregnancy-, birth-, and offspring-related health risks deserves greater exploration. Specifically, sexually active women who may become pregnant who use alcohol and smoke tobacco may experience negative impacts on fertility, as well as worse pregnancy outcomes and health outcomes for offspring^6^.

We conducted a secondary data analysis of the CHOICES Plus intervention trial data^7^ with our primary aim to characterize AEP and SEP risk by smoking status among patients being seen in primary care settings. We also analyzed indices of psychological distress (i.e. somatization, depression, and anxiety) by smoking status. Generally, we hypothesized that women who may become pregnant who smoke tobacco would drink alcohol more frequently and consume greater quantities, as well as report more lifetime substance use and experience greater psychological distress.

## METHODS

The CHOICES Plus RCT is registered on clinicaltrials.gov (NCT01032772). Briefly, CHOICES Plus was a 2-parallel-group design that allocated 261 participants in a 1:1 ratio to the intervention (n=131) or Brief Advice, with referral to community resources (n=130) between 2011 and 2013. Participants were recruited from 12 primary care clinics from a single, large public healthcare system in Houston, Texas, US. Women capable of becoming pregnant were eligible; however, gender identity was not assessed in this study. All participants were between aged 18–44 years, drinking more than 3 drinks/day or more than 7/week, sexually active, and not using effective contraception.

Study protocols were approved by IRBs at the University of Texas at Austin, Baylor College of Medicine, and Harris Health System. All participants provided informed consent, received compensation for study visits, and completed assessments in person with research staff at baseline and follow-up visits through 9 months post randomization. Participants completed the baseline assessment in the clinic with research staff prior to randomization and only baseline study data were analyzed for this manuscript, unless noted otherwise. Timeline follow-back methodology^8^ assessed alcohol and contraception use from 90 days prior to the baseline visit until the 9-month visit.

The baseline assessment included questions on participant characteristics and alcohol, tobacco, and illicit drug use, including the AUDIT^9^. Several other alcohol-related domains were assessed, including: readiness to change^10^, pros and cons for changing^11,12^, experiential and behavioral processes of change^13^, and temptation to drink and confidence to abstain^14-16^. Smoking status was assessed using multiple criteria. Specifically, self-report of any smoking on a 7-day point prevalence and/or cotinine readings >30 ng/mL on a NicAlert cotinine saliva assay kit were coded as smoking^17-20^. Psychological distress was assessed with the Brief Symptom Inventory 18 (BSI-18), comprising 3 subscales, taken from 18 self-reported items measuring somatization, depression, and anxiety symptoms^21^. Items on the BSI-18 have a 5-point rating scale (0=‘not at all’ to 4=‘extremely’). The BSI-18 manual considers t-score elevations ≥63, which represent the top 9% of normative respondents, to be indicative of significant scale elevation for each of the 3 subscales^22^. When 2 of the 3 subscales are ≥63, clinical follow-up is indicated^23^.

Descriptive data are provided by smoking status. All data analyses were performed in SAS, version 9.4 (Cary, NC, US) and all statistical tests were evaluated at α=0.05 (2-tailed) for significance. PROC GLM was used to compare continuous outcomes across smoking status and a chi-squared test (χ^2^) was used for current drug use (yes/no) comparisons by smoking status. For the BSI subscales, differences by smoking status were analyzed and significant differences were reported using T-scores^21^.

## RESULTS

Participants were primarily Hispanic (n=123; 47.1%) or non-Hispanic Black (n=109; 41.8%), and 69.3% (n=181) reported household incomes <$20000/year. Forty-five percent of participants (n=118) were currently smoking at baseline. Baseline comparisons by smoking status are reported in Table 1 with statistical significance testing.

**Table 1:** Baseline alcohol and substance use data of individuals who currently smoke compared to those who do not, who were recruited from 12 Primary Care Clinics in Houston, Texas, USA, between 2011 and 2013 for a randomized controlled trial testing an intervention to reduce substance-exposed pregnancies (N=261)

	*Current smoking (N=118) Mean ± SD*	*Non-smoking (N=143) Mean ± SD*
Drinking days per week	3.3 ± 2.0**	2.7 ± 1.8
Drinks/day on drinking days	5.8 ± 4.2	4.9 ± 4.2
≥4 drinks/day (past 90 days)	19.8 ± 24.7	16.6 ± 24.3
Greatest number of drinks in one day	10.1 ± 7.1*	8.2 ± 5.3
Importance: Drink less than risky^a^	6.0 ± 3.2	6.6 ± 2.8
Readiness: Drink less than risky^a^	6.7 ± 3.2	6.3 ± 2.8
Confidence: Drink less than risky^a^	6.3 ± 3.1***	7.4 ± 2.8
Number of different drugs used in lifetime	3.0 ± 2.0***	2.0 ± 1.5
URICA^b^: Readiness reduce alcohol	6.2 ± 3.0*	5.2 ± 2.9
Confidence to not drink alcohol	2.8 ± 0.9**	3.1 ± 1.0
Temptation to drink alcohol	3.0 ± 0.9**	2.6 ± 0.9
AUDIT total scores	12.1 ± 7.6*	9.8 ± 7.0
BSI: Anxiety (raw scores)	5.9 ± 5.6*	4.5 ± 4.9
BSI: Depression (raw scores)	6.8 ± 6.1	5.7 ± 5.5
BSI: Somatization (raw scores)	4.9 ± 4.8	4.1 ± 4.0

Raw scores are reported in this table for BSI subscales. PROC GLM (SAS, 9.4) was used to make all comparisons reported in this table and comparisons were evaluated at the p=0.05 level (2-tailed). AUDIT: alcohol use disorders identification test. BSI: brief symptom inventory. URICA: University of Rhode Island Change Assessment scale.

a Assessed via a 1–10 importance, confidence, readiness ruler to drink less than risky levels.

b Sum score from URICA scale calculated as (contemplation + action + maintenance) - precontemplation; range of possible readiness scores is -2 to 14.

* p<0.05.

** p<0.01.

*** p<0.0001.

Compared to participants who denied current smoking (n=143), participants who smoked reported significantly more drinking days per week (mean=3.3, SD=2.0 vs mean=2.7, SD=1.8 days, t=2.79, p<0.01), a higher mean greatest number of drinks in one day (mean=10.1, SD=7.1 vs mean=8.2, SD=5.3 drinks, t=2.47, p<0.05), and higher AUDIT scores (mean=12.1, SD=7.6 vs mean=9.8, SD=7.1, t=2.57, p<0.05). Participants who smoked were also more likely to report current drug use (66.1% vs 48.3%, χ^2^=8.37, p<0.01) and a greater number of (lifetime) illicit drugs used (mean=3.0, SD=2.0 vs mean=2.0, SD=1.5 days, t=4.32, p<0.0001), compared to participants who did not smoke. Furthermore, participants who smoked reported lower confidence to not drink alcohol (mean=2.8, SD=0.8 vs mean=3.1, SD=1.0, t= -2.72, p<0.01), less confidence to reduce risky drinking (mean=6.3, SD=3.1 vs mean=7.3, SD=2.8, t= -2.89, p<0.0001), greater temptation to drink (mean=3.0, SD=0.9 vs mean=2.6, SD=0.9, t=3.32, p<0.01), and, yet greater readiness to reduce alcohol use (mean=6.2, SD=3.0 vs mean=5.2, SD=3.0, t=6.72, p<0.05).

On psychological distress comparisons, all indices were greater for participants who smoked, consistent with experiencing more psychological distress. However, only the anxiety subscale was statistically significant (T-scores: mean=55.7, SD=12.1 vs 52.5, SD=11.0, t=2.2, p<0.05).

## DISCUSSION

This secondary data analysis re-examined data from a randomized controlled clinical trial that targeted alcohol and tobacco use in the preconception phase among women who may become pregnant. Specifically, we examined whether tobacco use was significantly associated with levels of alcohol and other substance use at baseline and associated with psychological distress at baseline. Alcohol and substance use at baseline served as proxies for potential AEP and SEP risk and we identified several indices of elevated AEP and SEP risk for individuals who also engaged in tobacco use, who were also more likely to report anxiety symptoms.

Individuals who smoked tobacco tended to report an extra day of drinking per week, as well as an additional two drinks/day on the day on which they drank the most. While both tobacco and non-tobacco users in this sample fell in the ‘hazardous or harmful’ alcohol use range on the AUDIT, those using tobacco tended to score 2 points higher, potentially translating into greater experience of alcohol-related problems. Prevalence of current drug usage was nearly 20% greater among individuals who reported current tobacco usage and individuals using tobacco tended to report using a greater number of drugs over their lifetime.

In the context of lower reported confidence to reduce risky drinking and greater temptations to drink, people who may become pregnant who use tobacco may need more intensive behavioral interventions to target alcohol and substance-related use and behaviors to reduce AEP and SEP risk. Importantly, participants who reported current tobacco use indicated greater readiness to reduce risky drinking, perhaps indicating a greater awareness of their higher alcohol use severity. This is encouraging and suggestive of opportunity and success for intervention with this population.

The greater level of anxiety symptoms reported by participants who smoked may suggest that anxiety is a symptom of increased tobacco, alcohol, and other substance consumption, or heightened anxiety may contribute to increased use of these substances, and/or the relationship may be bidirectional in nature. Our cross-sectional design limits greater inference with this sample but negative emotional experiences are a well-known trigger for substance use and anxiety would ideally be a clinical target of interventions designed to reduce SEPs.

### Limitations

This study was not without additional limitations. Given the rise in nicotine vaping among people of reproductive age^24^, it is important for future research to monitor if the identified trends similarly apply to those who vape, as these data were collected prior to recent vaping trends. Concerns about self-reported alcohol and other substance use data were partially mitigated by timeline follow-back methods and objective saliva cotinine assessments aided greater reliability regarding smoking status determinations. We also chose to retain a 0.05 alpha level to test for significance in this hypothesis-generating work – rather than apply a familywise error rate correction (e.g. Bonferroni) at this exploratory stage. Future research will determine which relationships replicate and improve on our work by exploring potential confounding variables and whether these results can be generalized to other countries.

## CONCLUSIONS

A significant proportion of pregnant women (5%) and those who may become pregnant (25%) engage in co-use of alcohol and tobacco or cannabis/other substances and tobacco^5^, which carries significant health risks for those who are pregnant and their children^6^. Our data demonstrated elevated alcohol and substance use risks for individuals also using tobacco, potentially requiring greater intervention intensity to eliminate and reduce health risks. Healthcare professionals should be aware of increased risks associated with tobacco and alcohol co-use, including heightened anxiety, and apply screening and brief interventions as indicated.

## Data Availability

The data supporting this research are available from the authors on reasonable request.
